# Intra-aneurysmal flow disruption after implantation of the Medina^®^ Embolization Device depends on aneurysm neck coverage

**DOI:** 10.1371/journal.pone.0191975

**Published:** 2018-02-06

**Authors:** Andreas Maximilian Frölich, Marie Teresa Nawka, Marielle Ernst, Isabell Frischmuth, Jens Fiehler, Jan-Hendrik Buhk

**Affiliations:** Department of Diagnostic and Interventional Neuroradiology, University Medical Center Hamburg-Eppendorf, Hamburg, Germany; Universitatsklinikum Freiburg, GERMANY

## Abstract

**Background and purpose:**

Flow disruption achieved by braided intrasaccular implants is a novel treatment strategy for cerebrovascular aneurysms. We hypothesized that the degree of intra-aneurysmal flow disruption can be quantified in vitro and is influenced by device position across the aneurysm neck. We tested this hypothesis using the Medina^®^ Embolization Device (MED).

**Methods:**

Ten different patient-specific elastic vascular models were manufactured. Models were connected to a pulsatile flow circuit, filled with a blood-mimicking fluid and treated by two operators using a single MED. Intra-aneurysmal flow velocity was measured using conventional and high-frequency digital subtraction angiography (HF-DSA) before and after each deployment. Aneurysm neck coverage by the implanted devices was assessed with flat detector computed tomography on a three-point Likert scale.

**Results:**

A total of 80 individual MED deployments were performed by the two operators. The mean intra-aneurysmal flow velocity reduction after MED implantation was 33.6% (27.5–39.7%). No significant differences in neck coverage (p = 0.99) or flow disruption (p = 0.84) were observed between operators. The degree of flow disruption significantly correlated with neck coverage (ρ = 0.42, 95% CI: 0.21–0.59, p = 0.002) as well as with neck area (ρ = -0,35, 95% CI: -0.54 –-0.13, p = 0.024). On multiple regression analysis, both neck coverage and total neck area were independent predictors of flow disruption.

**Conclusions:**

The degree of intra-aneurysmal flow disruption after MED implantation can be quantified in vitro and varies considerably between different aneurysms and different device configurations. Optimal device coverage across the aneurysm neck improves flow disruption and may thus contribute to aneurysm occlusion.

## Introduction

Intra-aneurysmal flow disruption achieved by intrasaccular implants composed of a braided metallic mesh is a novel treatment option for cerebrovascular aneurysms. The devices are designed to treat aneurysms with challenges to conventional coil embolization, e.g. those with a wide neck. A potential advantage of this device class over adjunctive techniques such as stent-assisted coil embolization is the lack of implanted material inside the parent vessel, which might reduce the risk of thromboembolic complications, alleviate the need for dual anti-platelet medication and allow use in acutely ruptured aneurysms [1]. Commercially available flow disruption devices include the Woven Endobridge Device (WEB, Microvention), the Artisse Device (Medtronic, Dublin, Ireland) and the Medina Embolization Device (MED; Medtronic, Dublin, Ireland). The MED combines the design of a detachable coil and an intrasaccular flow disruptor. The device consists of braided mesh petals oriented along the loops of the core wire. So far, four clinical reports describing case series treated with the MED are available [2–5]. Early clinical experience suggests that complete coverage of the aneurysm neck by the braided petals should be achieved to maximize the flow-disrupting effect of the device [3–5]. It has been suggested that incomplete neck coverage may predispose the aneurysm for recanalization due to insufficient flow disruption [3]. We tested the hypothesis that aneurysm neck coverage by the MED modulates flow disruption using patient-specific aneurysm models *in vitro*.

## Materials and methods

### Study design

This single-center study was approved by the local institutional review board (Ethik-Kommission der Ärztekammer Hamburg) with waived individual consent. Anonymized 3D rotational angiography (3D RA) data from ten aneurysms were retrospectively selected and used to manufacture patient-specific aneurysm models. The models were integrated into a flow circuit and MED placement was performed in vitro by two operators.

### Model fabrication

**Fig 1:**
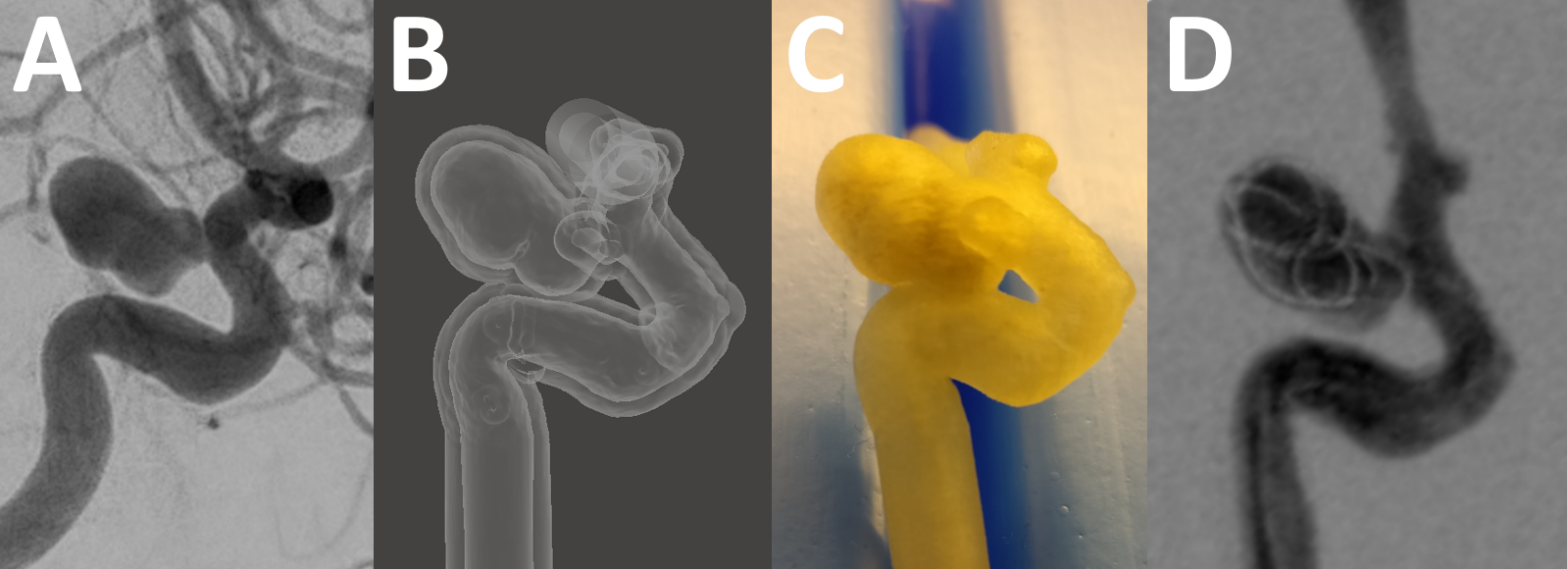
Model fabrication. A, Digital subtraction angiography from a patient with a left-sided internal carotid artery aneurysm originating at the posterior communicating artery origin. B, Hollowed surface data after postprocessing, before manufacturing. C, Photograph of the model after manufacturing. D, A single frame from high-frequency digital subtraction angiography in the model, obtained with a MED framer device deployed in the aneurysm sac.

3D RA data were acquired using an Allura Xper FD 20/20^™^ angiography system (Philips Healthcare, Best, The Netherlands) and processed using Analyze 11.0 (AnalyzeDirect, Inc., Overland Park, KS, USA) and CATIA V5 (Dassault Systèmes SA, Vélizy-Villacoublay, France) as previously described [6] in order to obtain volumetric surface files of a hollow aneurysm model suitable for additive manufacturing (Fig 1). Wall thickness of the hollow models was 1.0mm. Several model instances were produced by an external manufacturer (TBKO—Thomas Bengel Konstruktion + Prototypen, Meßstetten, Germany) using a commercially available machine (OBJET Connex, Stratasys Ltd., Eden Prairie, MN, USA) with an elastic, semi-transparent building material (TangoPlus FLX930 27 Shore A). Image data are available upon request.

### Procedural setup

Models were connected to silicone tubing and to a roller-type cardiovascular flow pump (Model Type 10-00-00, Stöckert Instrumente, Munich, Germany) to obtain pulsatile flow (pulse rate 60/min; flow rate 200mL/min). Fluid was heated to 37.0°C by a thermostat-controlled heating element. To approximate the viscosity of blood at this temperature, the system was filled with a mixture of 45% glycerol and 55% water to yield a dynamic viscosity of approximately 3.5 mPas [7]. A small amount of liquid soap was added to reduce friction. The system was placed in an Allura Xper FD 20^™^ angiography system (Philips Healthcare, Best, The Netherlands).

### Aneurysm embolization

Aneurysm models were treated by a two interventionalists over the course of four consecutive days. Operator 1 had 2 years experience of performing neurointerventions while operator 2 had 11 years experience. Both operators had prior laboratory, but not clinical experience with the MED. Access was obtained via a 6F guide and 0.021” microcatheter (Rebar 18, Medtronic, Dublin, Ireland). Framing MEDs in sizes from 4.0 to 9.0 mm were available (Medtronic, Dublin Ireland; distribution currently on hold). For each aneurysm, operators selected an appropriate framing MED size in consensus. Subsequently, operator 1 deployed and retrieved the MED inside the aneurysm four times to result in four different device configurations. After each deployment, flat-panel computed tomography (VasoCT, Philips Healthcare, Best, The Netherlands) and high-frequency digital subtraction angiography (HF-DSA; Aneurysm Flow, Philips Healthcare, Best, The Netherlands) were obtained. Then, an identical, new framing MED was introduced and operator 2 performed four deployments with accompanying imaging.

### In vitro image acquisition

3D RA was obtained with a 4s rotational acquisition, 220° rotation, 116 single frames at a frame rate of 29/s, 22cm detector field of view, 512 acquisition matrix. HF-DSA was obtained using a 19 cm field of view and a frame rate of 60/s for 420 total frames. 3 mL of contrast (Imeron 300, Bracco Imaging Deutschland GmbH, Konstanz, Germany) were injected by a coupled power injector with a flow of 1 mL/s via the 6F guide. The catheter tip was positioned approximately 15 cm upstream of the aneurysm. A projection was chosen to clearly discern the aneurysm and its neck from the parent vessel while avoiding vascular overlap in the feeding artery [8]. The flow pump was temporarily switched off to avoid movement artifact during VasoCT (220° rotation, 617 single frames at a frame rate of 30/s, 22 cm detector field of view and 512 acquisition matrix).

### Image reconstruction & analysis

HF-DSA was processed using commercially available software (Aneurysm Flow, Philips, Best, The Netherlands). The arterial segment used for the flow calculation was selected automatically by the software, in some instances requiring manual adjustment. Flow velocity fields were calculated in a region of interest including the aneurysm and adjacent parent artery. To measure the intra-aneurysmal flow velocity, a region of interest was placed by manually outlining the aneurysm sac. The mean intrasaccular flow velocity was then normalized to the parent artery flow by the software and used to calculate the mean aneurysm flow amplitude (MAFA) ratio as described by Pereira and colleagues [9]. The MAFA ratio represents the ratio of intra-aneurysmal flow velocity after and before MED implantation. As a more intuitively understandable variable, we calculated the flow velocity reduction (FVR) from the MAFA ratio as follows:

FVR(%)=(1−MAFA)*100

In addition, one observer manually scored intra-aneurysmal contrast opacification on three distinct phases of DSA: early phase (earliest image with full contrast filling of the parent artery), intermediate phase (maximal opacification of the aneurysm and parent artery) and late phase (earliest image after contrast washout in the parent artery). On each phase, intra-aneurysmal opacification was scored as 0 (none), 1 (faint) or 2 (full) in each of four quadrants of the aneurysm. The quadrants Q1-Q4 were defined with regard to the flow direction (Q1 basal proximal, Q2 basal distal, Q3 apical proximal and Q4 apical distal). The average score from the 4 quadrants was calculated for each phase.

**Fig 2:**
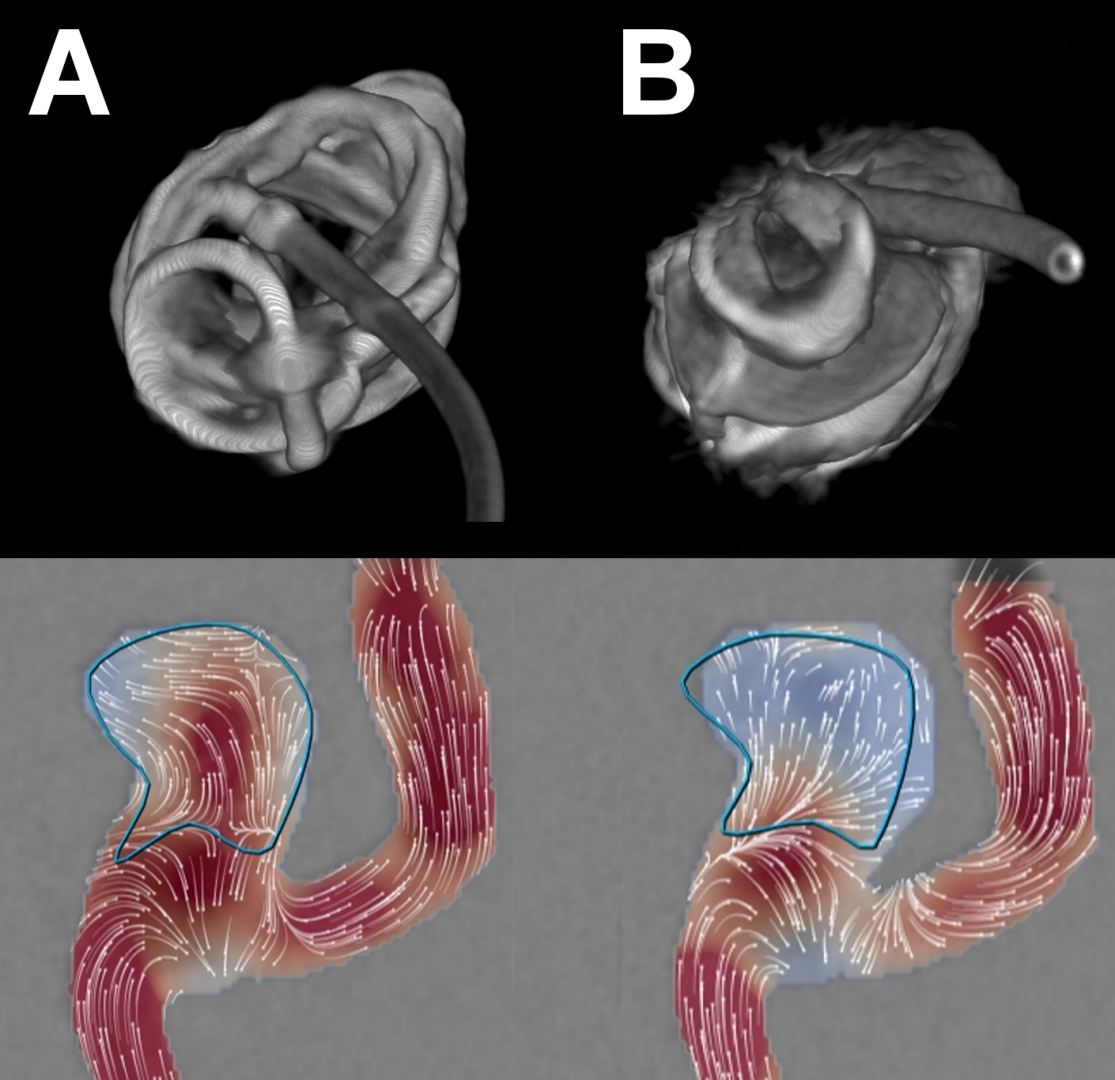
Neck coverage. Upper row shows volume reconstructions from VasoCT of two different MED configurations after implantation in the same aneurysm model (aneurysm 2), viewed from the direction of the aneurysm ostium. Incomplete neck coverage is appreciated in the first configuration (A, top), corresponding to moderate flow velocity reduction (41%) on high-frequency DSA (A, bottom). A different configuration showing better coverage of the neck by the MED petals (B, top) was accompanied by a higher degree of intra-aneurysmal flow velocity reduction (73%, B, bottom).

VasoCT was reconstructed with an isotropic voxel size (0.19 mm), 384 matrix and a stent-type kernel. Volume rendering was used to assess the individual braided mesh petals of the MED (Fig 2). With the option to overlay the 3D RA, two raters obtained an *en face* view of the aneurysm orifice and independently rated the deployment of MED petals across the aneurysm orifice on a 3 point Likert scale (“1 = insufficient coverage, less than 50% of orifice covered by petals”, “2 = partial coverage, 50–90% of orifice covered by petals”, “3 = complete or near-complete coverage, >90% of orifice covered by petals”). Median neck coverage ratings were obtained and dichotomized as ≥ 2 (favorable coverage) and <2 (unfavorable coverage). Aneurysm dimensions were measured on 3D RA.

### Statistical analysis

Baseline variables are described using descriptive statistics. One-way analysis of variance was performed to assess for differences in the achieved neck coverages across the different aneurysm morphologies. Spearman’s rank correlation was used to describe categorical variables. The intraclass correlation coefficient (ICC) for average measures was used to assess inter-observer agreement for the neck ratings. Step-wise multiple regression was used to assess the relationship of operator, neck coverage and neck area with FVR. Receiver operating characteristic curves where used to compare early, intermediate and late phase DSA-ratings for classifying the dichotomized neck coverage. All statistical analyses were performed using Medcalc 13 (MedCalc Software, Mariakerke, Belgium). The level of statistical significance was set at p = 0.05.

## Results

### Aneurysm model characteristics

Ten saccular cerebral aneurysms, all arising from the internal carotid artery, with maximum diameters ranging from 4.7 to 11.0 mm were included (Table 1). All aneurysm geometries were successfully reproduced, although residual support material sometimes had to be manually removed by flushing and slightly squeezing the models. Compared to our previous experience with models produced by fused deposition modeling [6], the current models manufactured by material jetting had a smoother surface, which allowed for much more realistic navigation with microwires and microcatheters. Still, both operators noted that interaction of the MEDs with the models’ walls appeared to be influenced by somewhat unrealistically high friction, which may have prevented a realistic “tumbling” movement of the developing framing coil. All eight individual MED deployments per aneurysm were successfully completed, resulting in 80 different total MED configurations recorded.

**Table 1:** Aneurysm characteristics.

Aneurysm #	Aneurysm location along ICA	Aneurysm dimensions (mm)	Neck area (mm^2^)	Dome-to-neck ratio	MED framer size (mm x cm)	Mean FVR (%, 95% CI)	Median neck coverage rating
1	Paraophthalmic	7.6x6.4x5.9	9,5	2,1	6 x 8	52.8 (40.9–64.6)	2
2	Supraclinoid	8.4x7.2x6.5	17,8	1,5	9 x 13	58.0 (46.3–69.7)	2
3	Supraclinoid	10.0x8.9x8.0	23,2	1,8	9 x 13	1.3 (-11.2–13.7)	1,5
4	Posterior communicating	11.0x6.0x6.5	8,0	3,4	8 x 10	27.1 (9.4–44.8)	2,5
5	Paraclinoid	7.7x8.1x8.1	14,4	1,8	8 x 10	4.3 (-10.9–19.4)	1,5
6	Infraophthalmic	7.4x8.4x6.7	14,2	2,5	8 x 10	58.5 (41.9–75.1)	2
7	Terminus	7.9x6.3x7.3	20,3	1,2	8 x 10	22.2 (-5.4–49.8)	1,5
8	Terminus	5.0x4.0x5.0	13,3	1,2	6 x 8	33.2 (13.4–52.9)	2,25
9	Posterior communicating	7.0x3.5x4.5	9,9	1,6	6 x 8	49.8 (39.5–60.0)	1,5
10	Supraclinoid	10.8x8.5x7.5	21,9	1,6	8 x 10	31.7 (19.8–43.7)	2

### Neck coverage and flow measurements

The neck area, dome-to-neck ratios and median neck coverage after MED implantation are reported in Table 1. We did not observe significant differences in the variance of achieved neck coverage ratings between aneurysms (p = 0,07). Interrater reliability for the neck coverage ratings was moderate (ICC for average measures: 0.70, 95% CI: 0.53–0.81).

HF-DSA was successfully recorded in all instances. On postprocessing, parent artery flow could not be calculated by the software in seven instances (distributed across four different aneurysms), reducing the total number of available flow measurements to 73 (see S1 Table for individual results). The mean MAFA ratio after MED placement was 0.66 (95%CI: 0.60–0.73), corresponding to an FVR of 33.6% (95%CI: 27.5–39.7%). The FVR achieved after MED implantation displayed considerable variability depending on the aneurysm (Table 1), ranging from a mean of only 1.3% (95% CI: -11.2–13.7%; aneurysm “3”) to a mean of 58.5% (95% CI: 41.9–75.1%; aneurysm “6”). We observed significant, moderate correlations between FVR and neck coverage (ρ = 0.42, 95% CI: 0.21–0.59, p = 0.002) as well as FVR and neck area (ρ = -0,35, 95% CI: -0.54 –-0.13, p = 0.024). On multiple regression analysis, both neck coverage (ρ_partial_ = 0.41, p = 0.0004) and neck area (ρ_partial_ = -0.28, p = 0.18) were independent predictors of FVR, while the operator was not. There was an overall weak fit of the regression model (multiple correlation coefficient: 0.51, R^2^ = 0.26).

**Fig 3:**
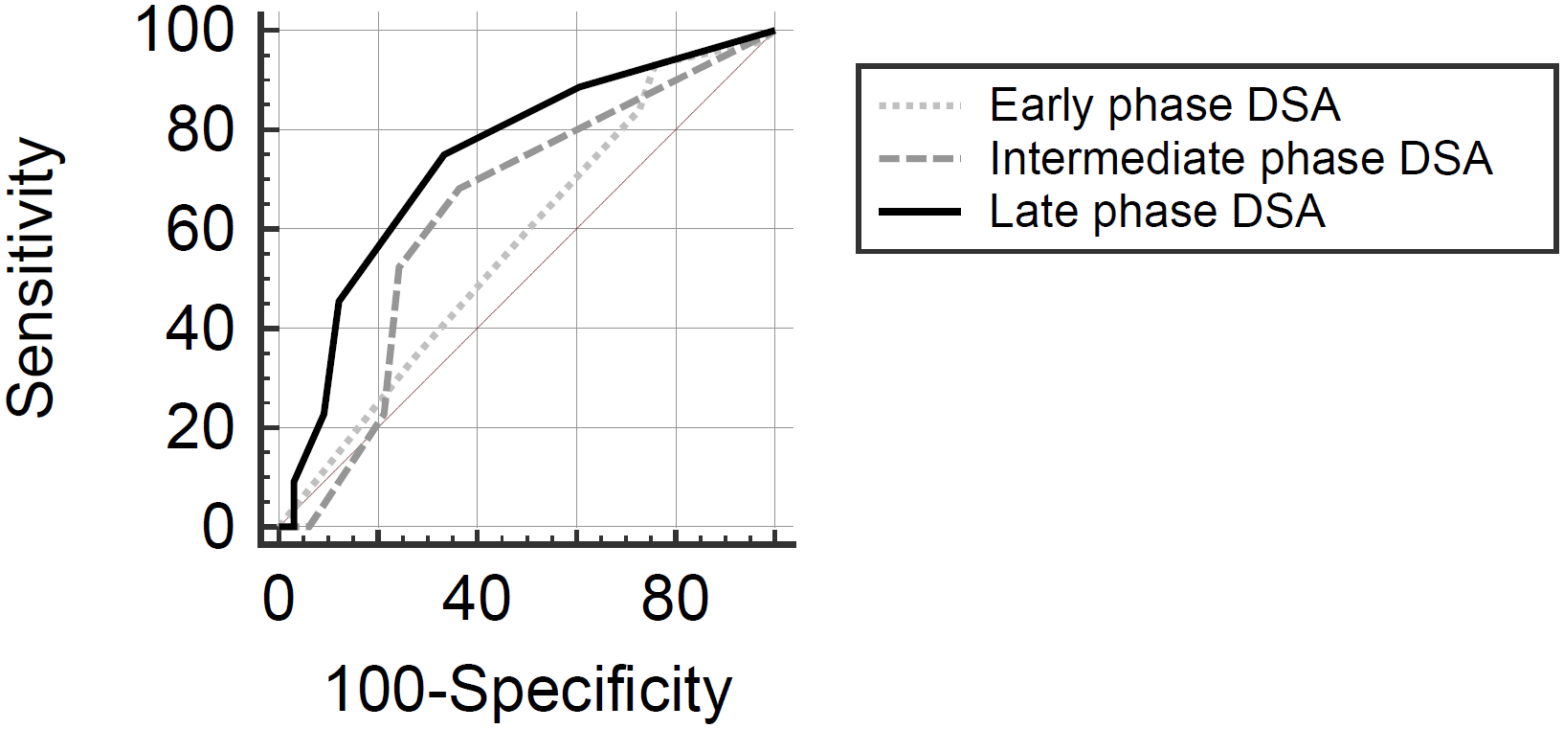
DSA-based rating of aneurysm opacification. Diagram shows receiver operating characteristic curves for classification of dichotomized neck coverage ratings based on early, intermediate and late phase DSA images. Intrasaccular stasis observed on late phase images had the highest area under the curve for discriminating favorable from unfavorable neck coverage.

DSA-based opacification scores showed a weak inverse correlation with neck rating on early phase (ρ = -0.23, 95% CI: -0.43 –-0.01, p = 0.045) and on intermediate phase (ρ = -0.29, 95% CI: -0.48 –-0.07, p = 0.010) and a moderate positive correlation on late phase images (ρ = 0.49, 95% CI: 0.30–0.65, p<0.001). On receiver operating characteristic curve comparison (Fig 3), late phase ratings provided the highest area under the curve (AUC) for predicting dichotomized neck coverage (AUC = 0,74, 95% CI: 0.63–0.84) compared to intermediate (AUC = 0.64, 95% CI: 0.52–0.74) and early phase (AUC = 0.58, 95% CI: 0.46–0.69).

## Discussion

This study demonstrates how the flow-altering effects of an intra-saccular flow disruption device can be assessed in a patient-specific in vitro environment. We observed a considerable variability of flow disruption after MED device implantation. This can in part be explained by the configuration of the MED petals across the aneurysm neck. The more tightly the braided petals cover the aneurysm neck and possibly overlap one another without intervening holes, the lower the porosity of the resulting orifical coverage, resulting in reduced inflow observed at HF-DSA. Interestingly, Jou recently demonstrated that HF-DSA can be used to similarly detect differences in porosity between different flow diverter stents in vitro [10], validating the utility of the technique.

Clinically, the variable degree of flow disruption may be one of the reasons governing whether a treated aneurysm will be occluded or will demonstrate persistent or recurrent filling, which has been observed with both the MED as well as the WEB device [2, 3, 5, 11, 12]. Our findings thus highlight the importance of adequate device positioning to cover the aneurysm neck. In fact, Turk et al. have used intrasaccular contrast media stasis on 6 frames/s digital subtraction angiography as a surrogate marker of satisfactory neck coverage after MED implantation [4]. Although we used a much higher frame rate and quantitative analysis, the principle approach of grading the intrasaccular flow response is the same and thus our in vitro data support application of their concept. In addition, our manual scoring of early, intermediate and late phase images showed that intra-aneurysmal contrast stasis on late phase images was the best discriminator of favorable neck coverage. This supports the concept that intra-aneurysmal stasis on DSA may be a useful biomarker to predict optimal neck coverage and aneurysm occlusion. Similarly, a recent analysis by Jeon et al. showed that the pattern of intra-aneurysmal contrast filling can predict progressive aneurysm occlusion after coil embolization [13]. Thus, the pattern and time course of residual contrast filling may better predict occlusion than the simple presence or absence of residual filling.

In addition, our data show that the degree of flow disruption not only depends on the device configuration after deployment, but also on the aneurysm itself. We have found a weak but significant detrimental effect of large aneurysm neck areas on FVR. This would suggest that adjunctive techniques, such as additional filling MED devices or coils, may be particularly valuable in larger, broad-based aneurysms to prevent residual or recurrent filling. In this context, Bhogal et al. have reported that aneurysms treated with only a single MED had a high chance of residual or recurrent filling and recommend against using a single device clinically [5]. Given the weak correlation we observed, other aneurysm-related factors beyond the scope of our analysis may also be important, such as the directionality of flow or the occurrence of turbulence.

Our study has several limitations. First, the in vitro setup only roughly approximates physiological flow conditions, which limits the generalizability of the results. We adjusted temperature, flow rate, pulse frequency and fluid viscosity, but other factors such as cellular blood components, platelet aggregation and plasmatic coagulation all affect the function of flow disruptors but are not reproduced in our model. Although anatomy was patient-specific, no patient-specific adjustments of internal carotid artery flow rate were performed. We furthermore did not test whether different flow rates or contrast injection speeds might have changed our observations, which would have drastically increased the time demand of the experiments. Importantly, the proprietary software used for HF-DSA analysis was designed for the use with flow diverter stents [9] (i.e. extrasaccular implants) and the accuracy of our results could have been affected by the presence of intrasaccular implants. However, since we only compared measurements with exactly one intrasaccular device, we would assume any such error to be systematic and not affect our comparisons. We did not correct the orientation of our models with regards to the gravitational field but positioned the models in such a way as to easily obtain a suitable working projection. Thus, the direction of gravity may have been different in our models compared to the original patient data. The behavior of devices during implantation is different in our models compared to reality (although we did not systematically assess this variable), particularly because of friction along the endoluminal surface. This may have affected the final device configurations. However, given that we obtained eight different device configurations for every aneurysm, observing a broad distribution of favorable and unfavorable neck coverages without significant differences between aneurysms, we would argue that these well represent the spectrum of clinically observable device configurations. Lastly, distribution of the MED is currently on hold, limiting the current clinical relevance of our findings. However, our observations should apply to future iterations of the device or other, similarly constructed flow disruption devices as a device class.

## Conclusion

The degree of intra-aneurysmal flow disruption after MED implantation can be quantified in vitro and varies considerably between different aneurysms and different device configurations. Optimal device coverage across the aneurysm neck improves flow disruption and may thus contribute to aneurysm occlusion.

## Supporting information

S1 Table Detailed results. (XLSX)
